# In vitro assessment of the accuracy of two intra-oral scanners for post space scanning in a fully digital workflow

**DOI:** 10.1186/s12903-025-05723-x

**Published:** 2025-03-19

**Authors:** Mennatallah Wahba, Reham Said ElBasty

**Affiliations:** 1 Fixed Prosthodontics Department, Faculty of Oral and Dental Medicine, Future University in Egypt, Cairo, Egypt; 2 Fixed Prosthodontics Department, Faculty of Dentistry, Cairo University, Cairo, Egypt; 3 Fixed Prosthodontics Division, School of Dentistry, New Giza University, Cairo, Egypt

**Keywords:** Accuracy, Intraoral scanner, Precision, Trueness

## Abstract

**Background:**

With the rapid advancements in computer-aided imaging, the potential for chairside fabrication of custom-made posts utilizing intraoral scanners may offer a reliable alternative to traditional physical impressions. Accordingly, this study aimed to evaluate the accuracy (trueness and precision) of two intra-oral scanners when different post space diameters were employed. Additionally, the scan depth of each intra-oral scanner (IOS) was assessed.

**Methods:**

An endodontically treated mandibular canine was inserted in a printed typodont model and prepared with two post-space diameters; a small one (1.5 mm) and a large one (2 mm). Polyvinyl siloxane impressions for the two post-space diameters were taken and then scanned with an extra-oral scanner to serve as reference scans. Each post-space diameter was scanned using CEREC Primescan (*n* = 8) and Medit i700 (*n* = 8) intra-oral scanners. Standard tessellation language (STL) files of all intra-oral and extra-oral scans were uploaded to a 3D matching program to evaluate trueness, precision, and post-space length difference. Statistical analysis was performed using different tests for parametric and non-parametric data. The significance level was set at *P* < 0.05.

**Results:**

Regarding the effect of the IOS and the effect of the post-space diameter, both Medit i700 and the small diameter groups using both scanners showed significantly higher root mean square (RMS) values when evaluating trueness. For precision, Medit i700 showed a significantly higher RMS value in the large diameter group. Medit i700 showed a significantly higher difference in post-space length than Primescan in both diameters. Primescan recorded a zero difference in the large diameter group compared to the reference scan.

**Conclusions:**

The IOS type and the post-space diameter influenced the scan accuracy and the depth of the scan. Accuracy results were superior when Primescan was implemented for scanning the large diameter post-space subgroup.

**Supplementary Information:**

The online version contains supplementary material available at 10.1186/s12903-025-05723-x.

## Background

Among the multiple challenges faced following root canal treatment is the substantial loss of coronal tooth structure, where oftentimes the use of a post is recommended to allow better retention to the core and the extra coronal restoration [1]. Though a prefabricated post can yield great outcomes in a case of adequate dentinal wall bulk, one of its main limitations is the discrepancy between the shape of the prefabricated post and that of the post space [2, 3] especially in cases where the root canal shows an oval shape, previous restorations with excessive preparations, incomplete root formation, or internal resorption [3–5]. Such scenarios would lead to less than optimum adaptation between the post and the canal wall with a gap formation leading to a non-uniform thick cement layer and consequently, the risk for structural discontinuity is heightened. Additionally, the increased polymerization shrinkage associated with the thick cement layer results in ultimately high internal stresses. All of which would possibly lead to fractures and post-debonding [3, 6, 7].

Despite the multiple approaches that have been introduced to minimize the discrepancy between the canal wall and the prefabricated post, the anatomic posts continue to serve as the gold standard for wide non-circular root canals displaying better fracture resistance, limited polymerization shrinkage stresses, and reduced gap formation during the cementation [3].

The advancement of computer-aided design/computer-aided manufacturing (CAD/CAM) technology in dentistry especially with the new generation of intraoral scanners (IOSs) having been validated as an efficient alternative to the traditional impression, alongside the currently available wide range of materials have made the production of custom-made posts easier, and more versatile [8–13].

Considering the raving race among manufacturers, it’s notable that clinicians, oftentimes, focus mainly on the speed and ease of use of the intra-oral scanner (IOS) together with some other practical aspects such as the possibility to omit the powder use for scanning, and the ability to export files without the need for paying any release fee. However, it is worth mentioning that the mathematical quality of the files obtained from the IOS is much more essential and influential in the success of the dental procedure [1] where accuracy is regarded as the principal mathematical feature an IOS should have [14–18].

Accuracy is defined as the sum of both trueness and precision [15–17]. According to the ISO international standard number 5725, trueness is the ability of a measurement to match the actual value of the quantity being measured. Alternatively, precision is the ability of a measurement to consistently repeat a particular measurement. Hence, the ideal IOS should be able to reproduce the surface of the scanned object as exactly as it is granting consistent and repeatable results with no deviations when the same object is scanned [15, 16, 19].

Determining the trueness of an IOS, a reference model onto which the intra-oral scans are superimposed is needed. On the other hand, for the sake of evaluating the precision of an IOS, multiple scans of the same object are being captured, and saved. It’s then through reverse-engineering software, that they are overlapped together to detect the deviation between them. High precision is guaranteed when minimal deviations between the models are achieved [15, 16, 20].

Through the past few years, a vast number of manufacturers have introduced different IOSs with different scanning technologies to the dental market. Nevertheless, according to the manufacturers’ claims, none of those IOSs, except for the Primescan and Medit i700, have the capability to scan depths greater than 16 mm. These two systems are capable of scanning deeper spaces, up to 20 mm and 23 mm, respectively [21, 22]. With very limited data being available to prove such claims, this study was designed aiming at examining the scanning accuracy (trueness and precision) of the 2 IOSs when different post-space diameters were employed. Additionally, the depth of the scan each IOS can reach was assessed.

The null hypotheses were that neither the type of the IOS nor the post-space diameter would affect the accuracy or scan depth of the scanner.

## Methods

Before the following study was carried out, it was ethically approved by the ethics committee of the Faculty of Dentistry, Cairo University (161222) since a caries-free human mandibular canine extracted for therapeutic reasons was used in the study.

A caries-free human mandibular canine having a root length of 16 mm and a crown length of 10 mm was chosen for our study. The anatomic crown of the mandibular canine was removed, using a high-speed diamond disk under water coolant, leaving only 3 mm above the cemento-enamel junction. The root canal was chemico-mechanically prepared where ProTaper Next rotatory system (Dentsply-Maillefer, Ballaigues, Switzerland) was used up to size X4. 2 ml of 2.5% NaOCl was utilized for chemical irrigation between each file and finally, a 17% EDTA was applied as a final rinse for one minute. The root canal was rinsed with distilled water for one minute and paper points were used to dry the canal before obturation. The canal was obturated with X4 gutta-percha (META BIOMED CO.LTD, Korea) and AdSeal resin sealer (META BIOMED CO.LTD, Korea) using modified single cone technique, and the access cavity was sealed with a temporary filling (Coltsol F, Coltene, Switzerland). The obturated tooth was stored in distilled water at 37 °C.

**Fig. 1:**
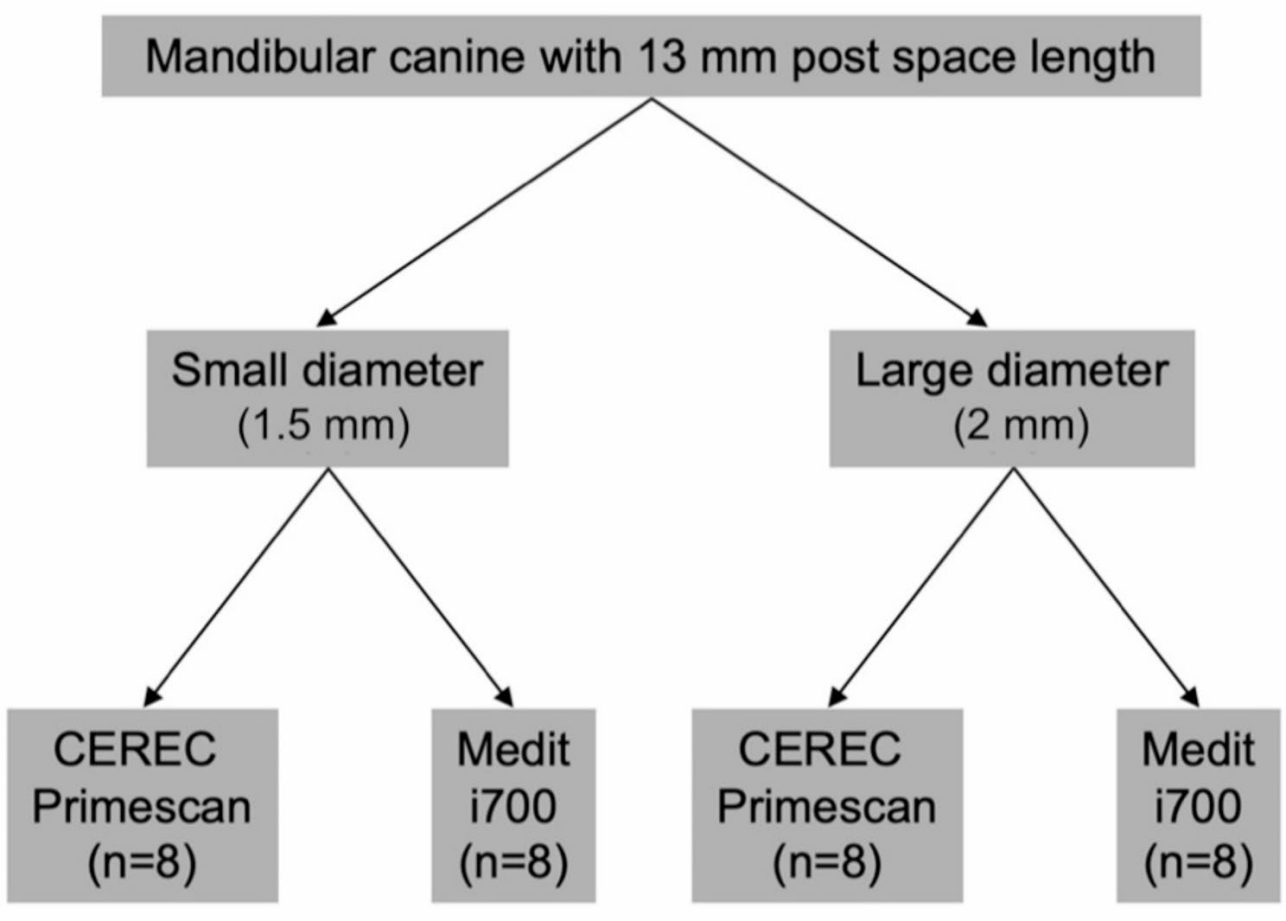
Flow scheme for sample grouping

According to the pre-determined study design, the canine was prepared with two post space diameters: a small diameter group (1.5 mm) and a large diameter group (2 mm). Each group was scanned using CEREC Primescan ‘software version 5.2.6’ (Dentsply Sirona; Bensheim, Germany) and Medit i700 Wireless ‘software version 3.0.6’ (Medit Corp. Seoul, Republic of Korea). A prior power analysis was performed based on a previous study [11] using the G*Power analysis software (v.3.1.9.7). The minimum total sample size was determined to be 32 scans (Fig. 1).

Subsequently, post space preparation was initiated where Peso drills (Mani, Italy) were first used to remove the gutta percha leaving an apical seal of 6 mm and thus creating a post space of 13 mm length. For the small diameter group, a 1.5 mm diameter yellow drill (Galssix Plus, Nordin) was used for canal enlargement.

**Fig. 2:**
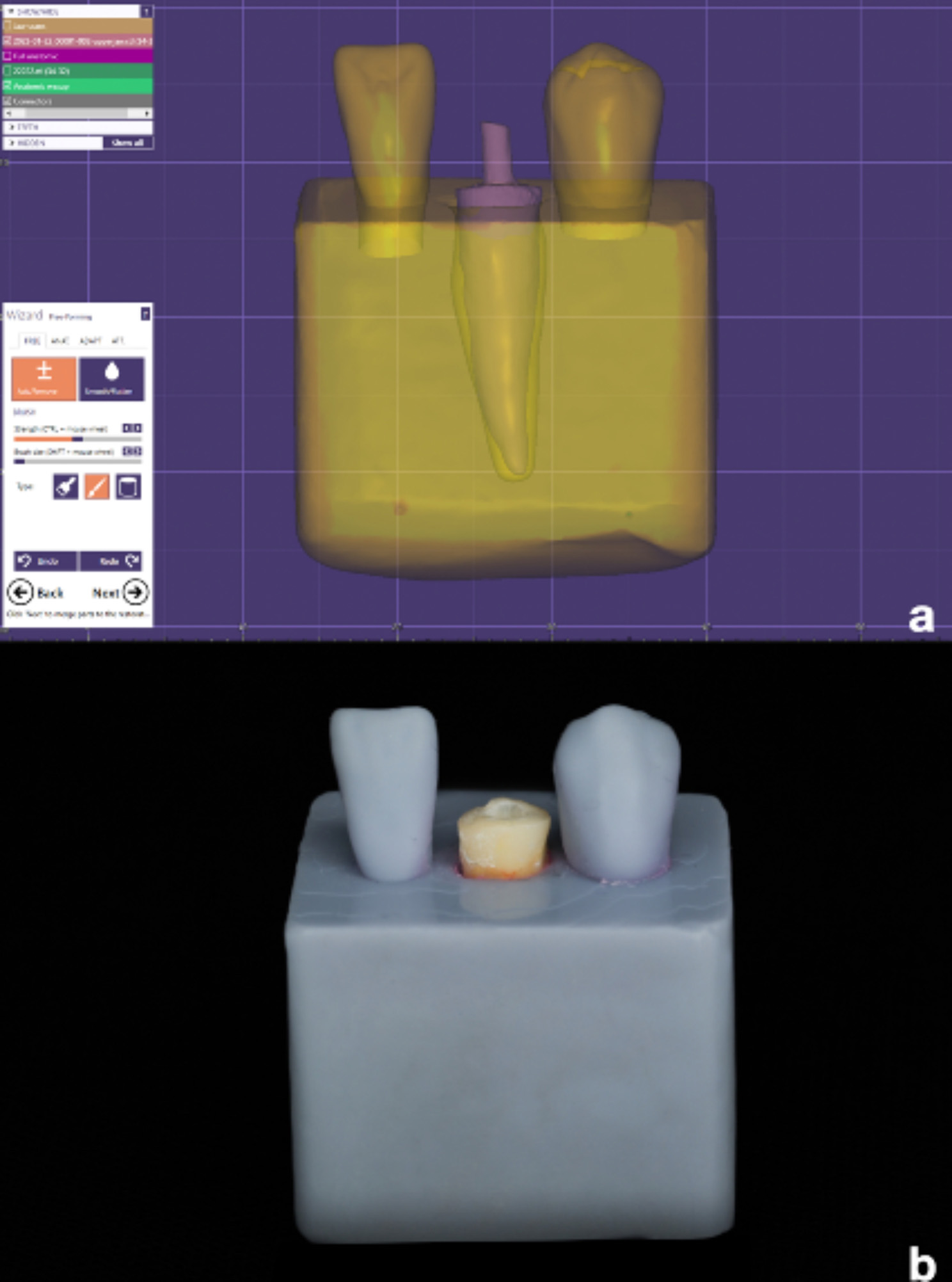
A custom 3-D printed typodont model featuring the mandibular lateral incisor, mandibular canine, and mandibular 1st premolar: (**a**) Software design of the model (**b**) The 3-D printed model with the mandibular canine in place

**Fig. 3:**
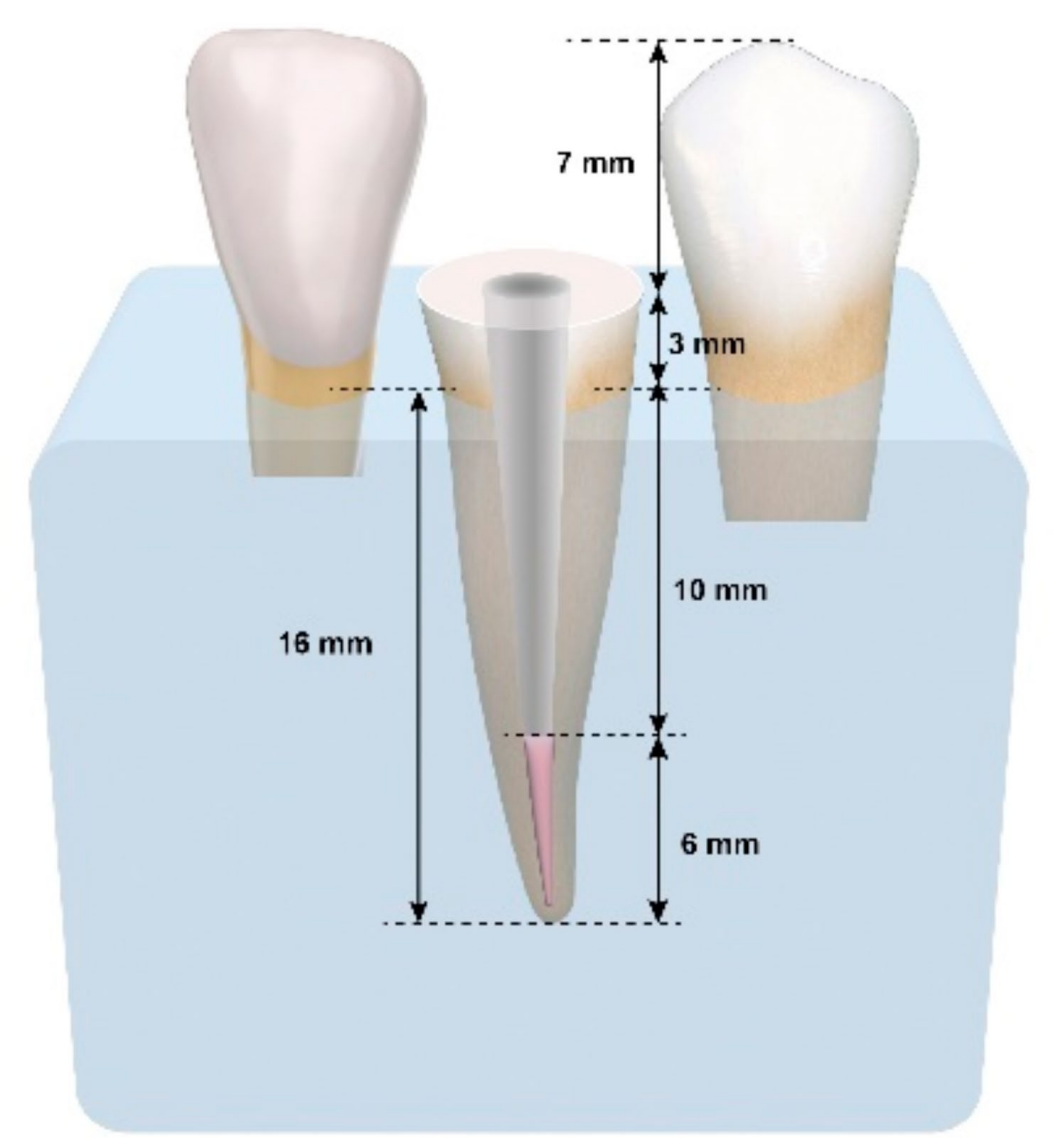
Diagrammatic drawing of a custom 3-D printed typodont model featuring the mandibular lateral incisor, mandibular canine, and mandibular 1st premolar

For proper scanning or impression taking in conditions simulating the clinical set-up [23, 24], a custom 3-D printed typodont model featuring the mandibular canine socket together with its two adjacent teeth; the mandibular lateral incisor and mandibular 1st premolar was designed (2.2 Valletta Exocad software) and printed out in resin (MODEL, Proshape Digital Solutions) using 3D printing technology (Creality, HALOT-SKY). After the canine root was checked for fit inside the socket of the printed model ensuring that only 3 mm of the tooth are extruding coronal to the base, the canine tooth was glued to the socket using cyanoacrylate resin (Amir Alpha) allowing a 7 mms of dead space between the butt joint of the mandibular canine and the incisal edge/cusp tip of the adjacent teeth (Figs. 2 and 3). Accordingly, a scanning depth of 20 mm was facilitated and checked using a rotary file adjusted to 20 mm via a rubber stopper.

**Fig. 4:**
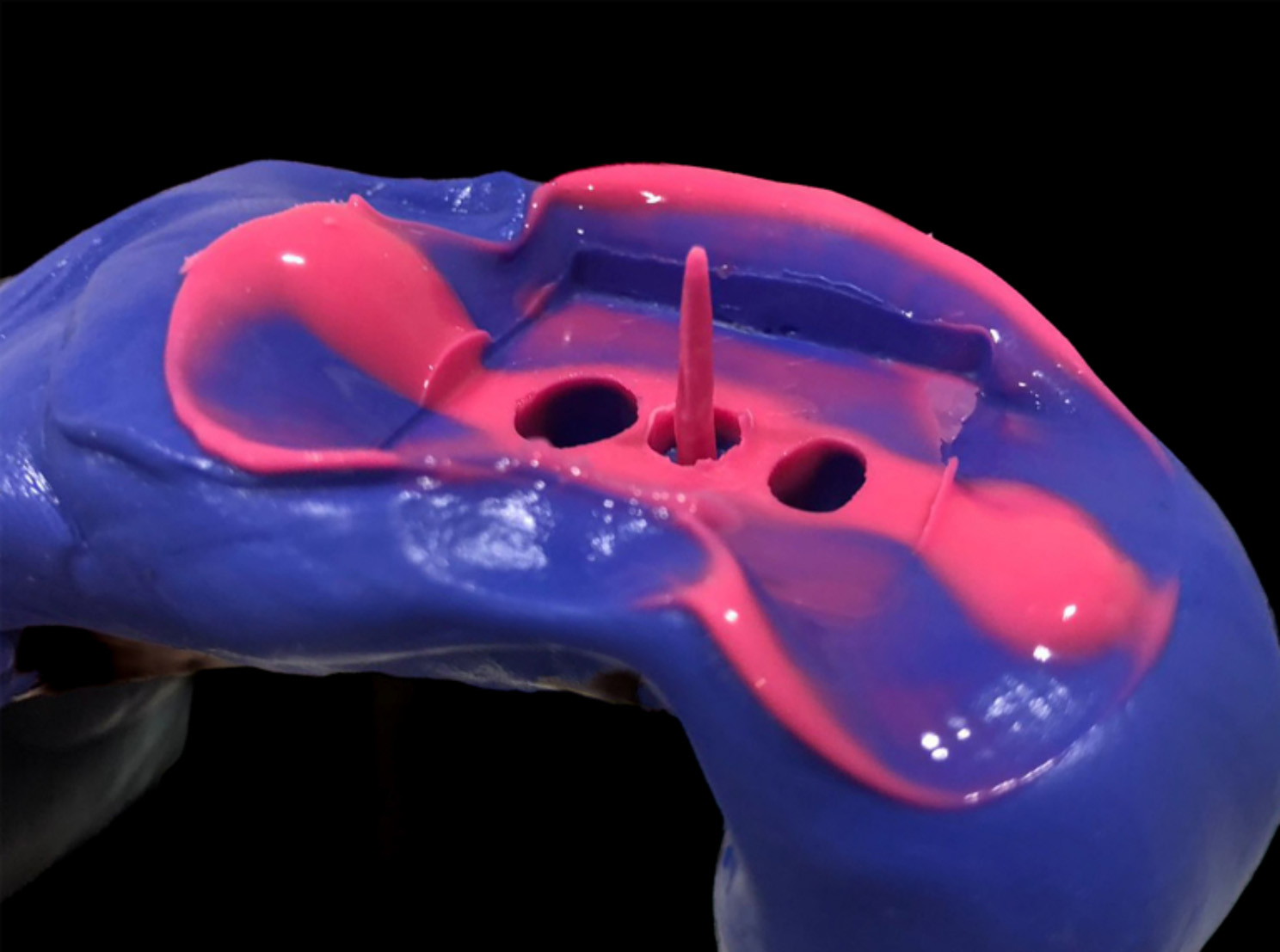
A conventional polyvinyl siloxane impression of the post space

Since a reference scan was mandatory for trueness measurements, a conventional impression of the post space was taken using putty and light-body polyvinyl siloxane impression material (Hydrorise, Zhermack, Italy) (Fig. 4). Using a 40 no Lentulo spiral (VDW; Munich, Germany), some of the light-body impression material was introduced inside the post space, then a customized wooden toothpick was stabilized in the center. A sectional stock tray with putty impression material was seated over the whole typodont model. When complete material polymerization was ensured, the impression was pulled out and the length of the post space impression was checked with a rotary file adjusted to 20 mm. The silicone impression was subsequently scanned with an extra-oral scanner (inEos X5; Dentsply Sirona; Bensheim, Germany), and a standard tessellation language (STL) data was recorded as a reference scan.

According to the predetermined sample size, scanning of the post space was executed 8 times using each of the two IOSs; CEREC Primescan and Medit i700 Wirelesss. Scanning was performed by the same investigator “E.R” with 10 years of experience. For scanning the model, the operator adopted the scanning pathway recommended by the manufacturer. The post space was scanned from different angles by tilting the scanner head to maximize the captured data. Each IOS was calibrated before scanning following the manufacturer’s guidelines. The data were recorded and saved as STL files to be compatible with the 3D matching program (Geomagic Control X 2020; 3D Systems; Rock Hill, SC, USA).

For the purpose of creating the larger diameter group, the same post space was enlarged using the same previously employed post drills system. Larger diameter drills were employed where the 1.8 mm diameter (red) drill, followed by the 2.0 mm diameter (blue) drill were utilized following the manufacturer’s instructions. The same steps for physical impression taking and intra-oral scanning were repeated after canal enlargement. However, the tooth was stored in 37 °C distilled water in between different steps to avoid dehydration.

All STL data sets were uploaded into a 3D evaluation software program (Geomagic Control X; 3D Systems) where the desktop scans of the post-space impression of both diameters were set as reference data, while the intra-oral scans were set as measured data. For each group, 3D segmentation of the model was performed to split the model into 2 parts; that of the neighbouring teeth and that of the prepared post-space. The part of the unprepared neighbouring teeth was employed for the purpose of superimposition between the models using the initial alignment and best-fit algorithm. Such step was performed to remove any irrelevant data that could affect the measurements such as the model base and the teeth limiting only the measurements to the area of interest which is the post-space. On the other hand, the part comprising the prepared post-space was used for the 3-D comparison between the models.

The STL files of the measured intra-oral scans were superimposed with the reference STL files for all groups for trueness measurement. However, for precision evaluation, intra-oral scan STL data sets of each group and captured by the same IOS were superimposed together.

**Fig. 5:**
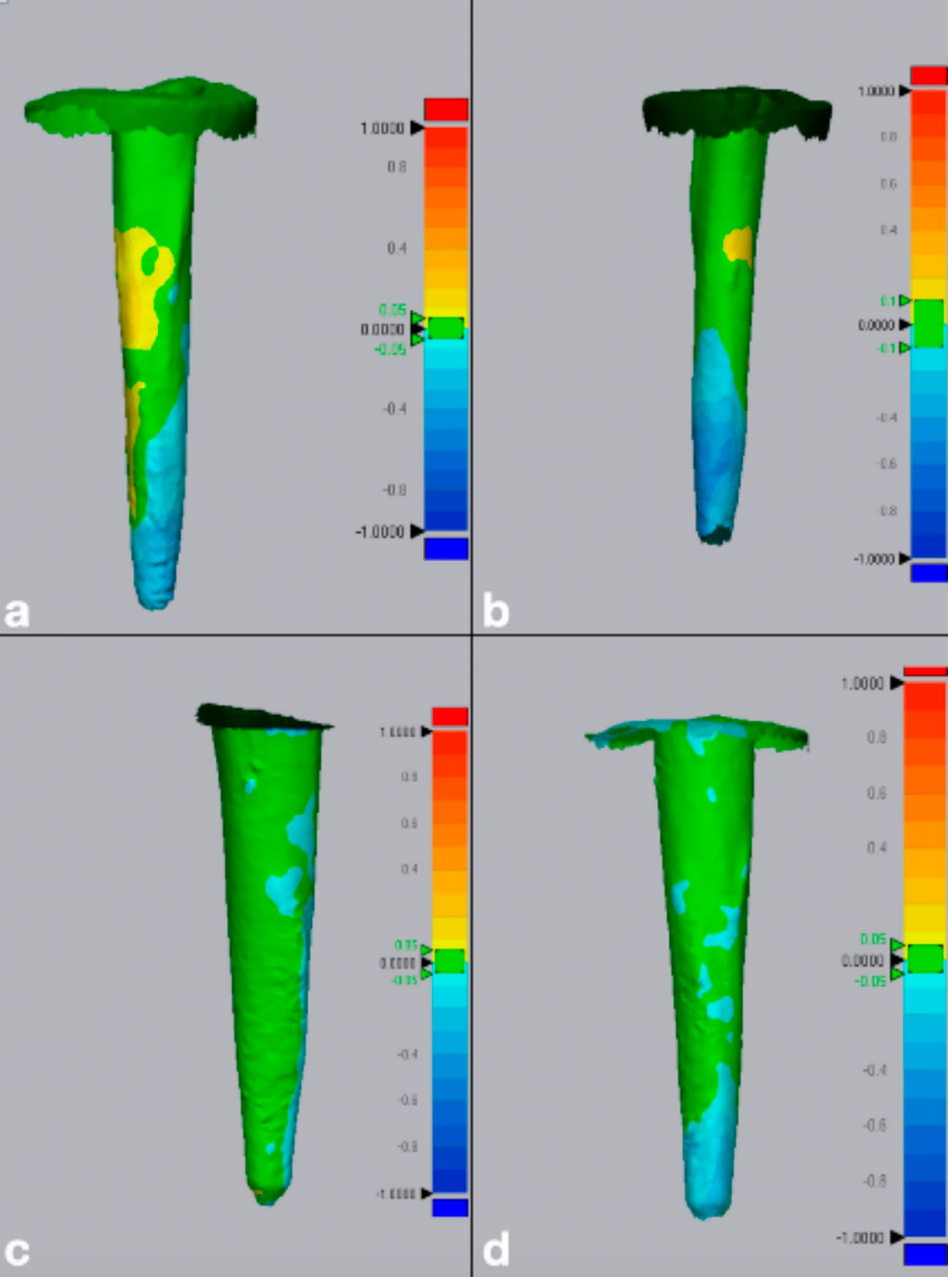
Representative color map images for trueness assessment. Range of deviation color coded from − 100 mm (blue) to + 100 mm (red): (**a**) Small diameter post space scanned with Prime Scan IOS (**b**) Small diameter post space scanned with Medit i700 IOS (**c**) Large diameter post space scanned with Prime Scan IOS (**d**) Large diameter post space scanned with Medit i700 IOS

**Fig. 6:**
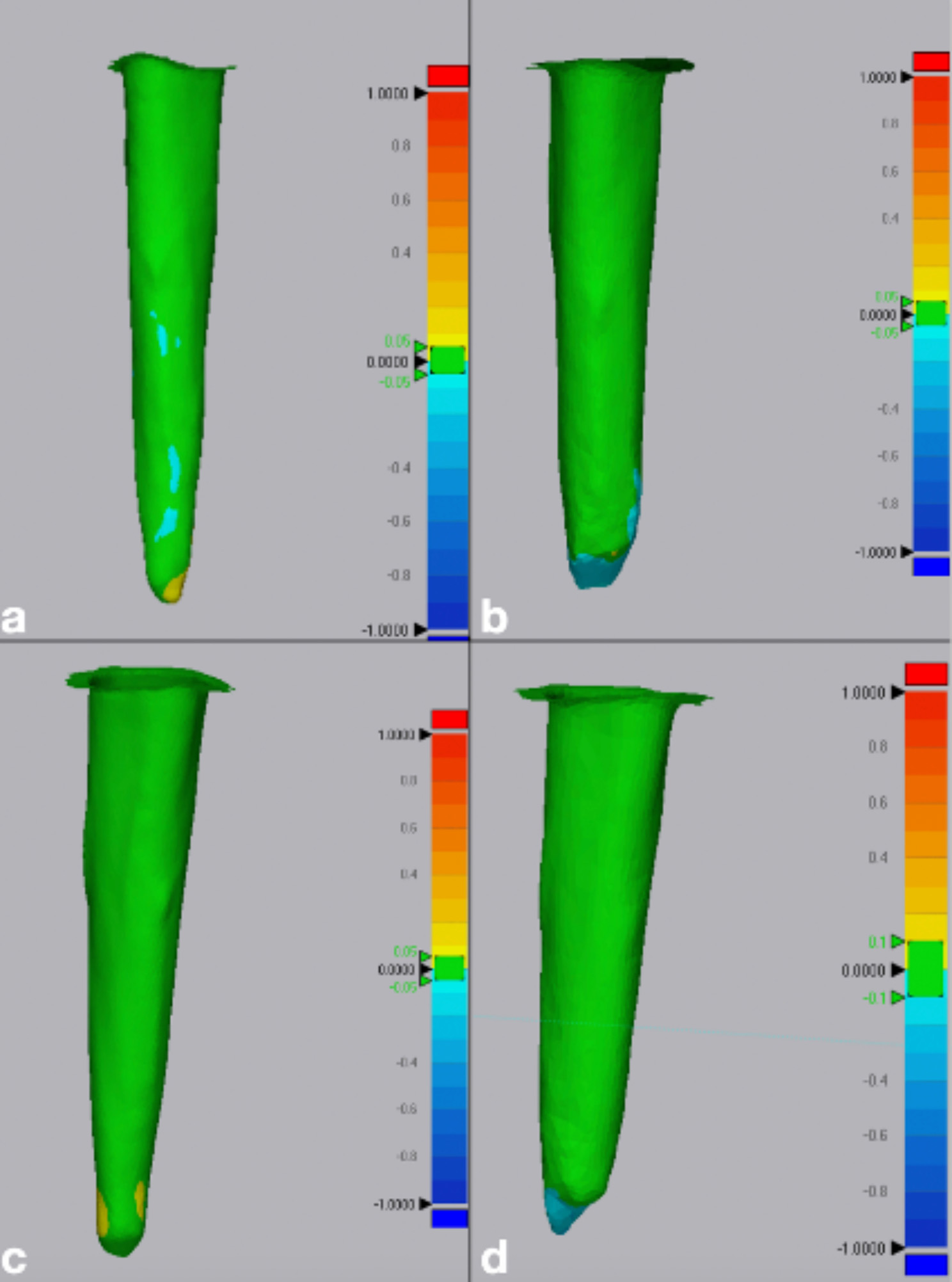
Representative color map images for precision assessment. Range of deviation color coded from − 100 mm (blue) to + 100 mm (red): (**a**) Small diameter post space scanned with Prime Scan IOS (**b**) Small diameter post space scanned with Medit i700 IOS (**c**) Large diameter post space scanned with Prime Scan IOS (**d**) Large diameter post space scanned with Medit i700 IOS

Discrepancies between the reference and measured data were calculated as the root mean square (RMS) values [12, 25]. The software automatically calculated the values for the evaluation of trueness, and the data were recorded for statistical analyses where a lower RMS value signifies higher trueness and vice versa. On the other hand, the RMS values between each set of scans of the same IOS were calculated for measurement of precision. A color map representing visual deviation [26, 27] in the region outside this range was used where the deviation range was being color coded from − 100 mm (blue) to + 100 mm (red) (Figs. 5 and 6).

**Fig. 7:**
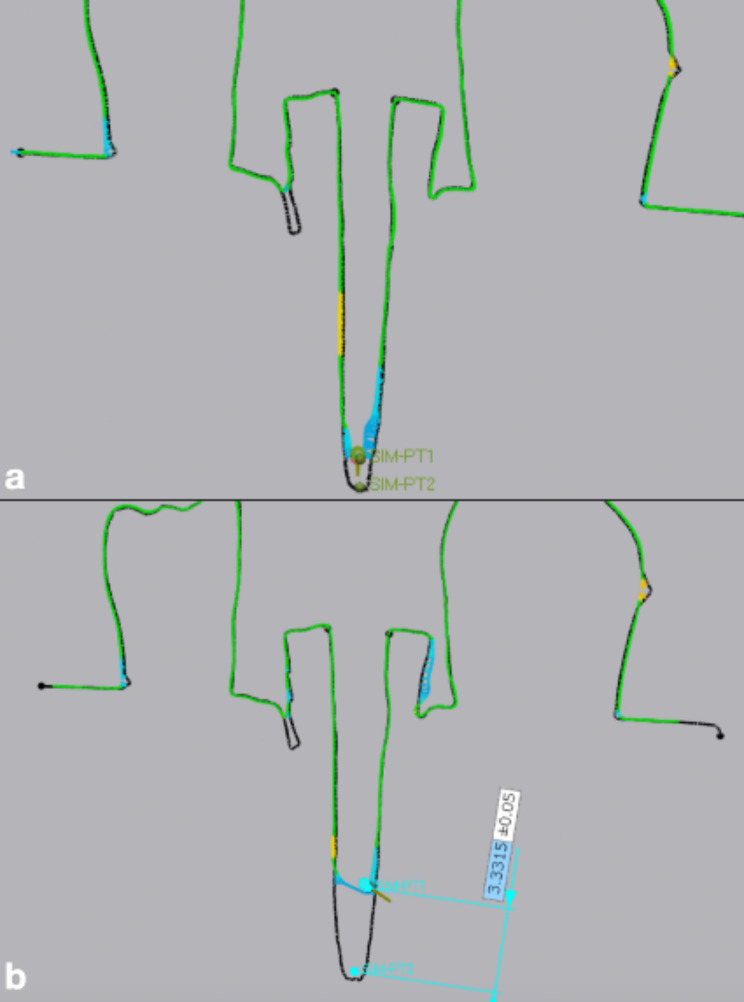
Assessment of the difference in post-space depth acquisition: (**a**) Small diameter post space scanned with Prime Scan IOS (**b**) Small diameter post space scanned with Medit i700 IOS

**Fig. 8:**
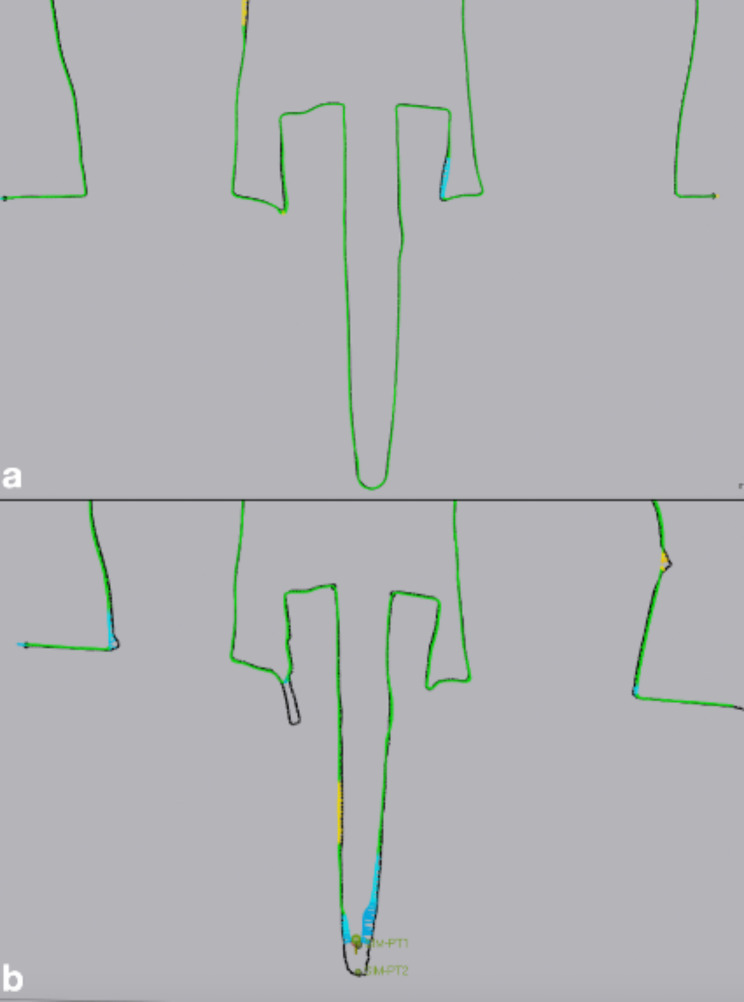
Assessment of the difference in post-space depth acquisition: (**a**) Large diameter post space scanned with Prime Scan IOS (**b**) Large diameter post space scanned with Medit i700 IOS

In order to compare the difference in post-space depth acquisition, simulated comparison points were set at the deepest points of the reference and measured scans followed by measuring the distance in mm between the comparison points (Figs. 7 and 8).

Numerical data were explored for normality by checking the distribution of data and using Kolmogorov-Smirnov and Shapiro-Wilk tests for normality. Both trueness and difference in post space depth data showed parametric distribution. On the other hand, precision data showed non-parametric distribution. Data were presented as mean, standard deviation (SD), median and range values. For non-parametric data, Wilcoxon signed-rank test was used to compare between the two IOSs. Mann-Whitney U test was implemented for the comparison between the small and large diameters groups. For parametric data, repeated measures ANOVA test was used for the comparison between the two IOSs as well as the two post space diameters. Bonferroni’s post-hoc test was used for pair-wise comparisons when ANOVA test was significant. The significance level was set at *P* < 0.05. Statistical analysis was performed with IBM SPSS Statistics for Windows, Version 23.0. Armonk, NY: IBM Corp.

## Results

Having a look on the trueness values, it was clear that in both small and large diameter groups, Medit i700 showed statistically significantly higher RMS values (0.119 and 0.062 respectively) than Prime scan (0.1 and 0.039 respectively). When considering the diameter effect, with the use of Medit i700 or Primescan, small diameter groups showed statistically significantly higher RMS values (0.119 and 0.1 respectively) than large diameter ones (0.062 and 0.039 respectively) (Table 1).

**Table 1:** Descriptive statistics and results of repeated measures ANOVA test for comparison between trueness values (RMS) of the two scanners and the two diameters

Diameter	Medit i700		Primescan		*P*-value	Effect size (Partial Eta squared)
	Mean	SD	Mean	SD		
Small	0.119	0.006	0.1	0.005	< 0.001*	0.84
Large	0.062	0.007	0.039	0.004	< 0.001*	0.885
*P*-value	< 0.001*		< 0.001*			
*Effect size (Partial Eta squared)*	0.955		0.981			

*: Significant at *P* < 0.05

Moving on to the precision results, results revealed no statistically significant difference between the two scanners (*P*-value = 1) in the small diameter groups. Yet, Medit i700 showed a statistically significantly higher RMS value (0.037) than Primescan (0.017) in the large diameter groups (Table 2).

**Table 2:** Descriptive statistics and results of Wilcoxon signed-rank test for comparison between precision values (RMS) of the two scanners and mann = whitney U test comparison between the two diameters

Diameter	Medit i700		Primescan		*P*-value	Effect size (d)
	Median (Range)	Mean (SD)	Median (Range)	Mean (SD)		
Small	0.036 (0.026, 1.445)	0.241 (0.531)	0.045 (0.022, 0.049)	0.042 (0.009)	1	0
Large	0.037 (0.028, 1.489)	0.242 (0.549)	0.017 (0.016, 0.025)	0.019 (0.004)	0.018*	3.053
*P*-value	0.482		0.003*			
*Effect size (d)*	0.726		2.802			

*: Significant at *P* < 0.05

Although there was no statistically significant difference in the precision values (*P*-value = 0.482) between the small and large diameters groups when Medit i700 was used, Primescan small diameter group displayed statistically significantly higher RMS value (0.045) than the large diameter one (0.019) (Table 2).

Regardless of both trueness and precision values, the difference in post-space depth captured compared to the reference scan was also assessed. Medit i700 showed a statistically significantly higher difference (*P*-value < 0.001) in post-space depth than Primescan in both diameters. Additionally, the small diameter groups scanned with both scanners revealed statistically significantly higher differences (*P*-value < 0.001) in post-space length than the large diameter groups. It was obvious through the results that in the large diameter group, Primescan was able to capture the full length of the post space with zero difference compared to the reference scan (Table 3).

**Table 3:** Descriptive statistics and results of repeated measures ANOVA test for comparison between difference in post space length values (mm) of the two scanners and comparison between the two diameters

Diameter	Medit i700		Prime scan		*P*-value	Effect size (Partial Eta squared)
	Mean	SD	Mean	SD		
Small	3.46	0.269	1.252	0.234	< 0.001*	0.953
Large	2.199	0.332	0	0	< 0.001*	0.953
*P*-value	< 0.001*		< 0.001*			
*Effect size (Partial Eta squared)*	0.833		0.942			

*: Significant at *P* < 0.05

## Discussion

With accuracy being a key factor for the success of any fixed dental restoration, and given the complexity of the post space, the ability of the IOS to capture and produce an accurate digital impression is questioned. Bearing in mind the ultimate role played by a perfect impression in the precise adaptation of a custom-made post, conventional physical impression of post space has remained the gold standard for years [23, 28]. Nevertheless, the use of such conventional impression materials and techniques has been reported to result in multiple difficulties and inconveniences from the operator’s and the patient’s standpoint [28, 29].

Meanwhile and with the ongoing progress taking place in the technology behind the digital dentistry, various IOSs based on different scanning technologies and softwares have been launched in the market, yet only Primescan and Medit i700 can reach scanning depths up to 20 mm and 23 mm respectively according to the manufacturers’ claims [21, 22]. Hence, authors designed that study to examine the scanning accuracy as well as the depth of scan of those two IOSs implementing different post-space diameters to simulate different clinical conditions of variable root diameters. With the canine being the common tooth to show long roots with ovoid canal shape that could possibly call for a custom fabricated post, a mandibular canine was used in the study and the post-space length was set to 13 mm and the scanning depth was 20 mm.

However, the null hypotheses for the current study were rejected since both the IOS type and post-space diameter had a significant effect on the trueness and precision of the scans as well as the scan depth of the scanner.

Regarding the trueness results, it was quite evident that for both small-diameter and large-diameter post spaces, Primescan IOS showed lower RMS values in comparison to Medit i700. Such better performance regarding the accuracy of Primescan compared to Medit i700 was confirmed previously by Dupagne L et al. [24] where Medit i700 displayed higher mean measurement error at different depths.

Numerous studies in the literature have reported an enormity of causes that would likely impact the accuracy of the scans. Among those factors are the complexity of the preparation anatomy, the limited angle of the scan, the depth of field, and the type of scanner [14, 24, 30, 31]. Dupagne L et al. as well as Park MJ et al. [24, 30] in different studies have affirmed that the depth of field of the IOS could most commonly affect the trueness claiming that scanners with shallower depth of field couldn’t possibly capture different surfaces with a sharp focus.

Moreover, Cui N et al., Dupagne L et al., and Park MJ et al. [18, 24, 30] have shown through their studies that the image acquisition method employed by different scanners greatly influenced the accuracy of the scan and consequently the relative amount of error. Having stated that, it’s worth mentioning that the measurement technique employed by the Primescan IOS is referred to as optical high-frequency contrast analysis [13]; a combination of confocal microscopy and fringe light projection, which may have an influence on the accuracy compared to sole confocal microscopy. On the other hand, Medit i700 utilizes the active triangulation technology [24].

Though Park MJ et al. [30] have stated that the active triangulation method of scanning resulted in more accurate scans compared to other scanning methods, yet such finding was not in accordance with our results since different intraoral scanners were used in the two studies. Moreover, Logozzo S et al. [32] have reported that the laser and/or camera occlusion resulting from the inaccessibility of light beam or scanner camera to scanned object would certainly influence the accuracy of the scans obtained through IOS based on active triangulation technology. However, it must be mentioned that it’s still not conclusively clear which scanning principle is the most effective according to the currently available data [30].

It’s worth mentioning here as well that the post space diameter showed a significant effect on the trueness of both IOSs used in the study. It was clear that the larger diameter groups displayed lower RMS values as well as less discrepancies in post space depth compared to the reference scan with both scanners. Such results agree well with Pinto A et al. [23] who demonstrated in a previous study that an IOS was capable of capturing comparable post space depths to conventional impression only when the post space entrances were made wider. They referred such outcome to the greater amount of scanning light being capable of entering the post space when larger entrance was allowed.

On the flip side, when precision results were considered, it was evident that the lowest RMS values were achieved with the Primescan scanning the large diameter group.

Upon assessing the post space depth, Primescan IOS showed higher capability to reach a greater depth together with an accuracy level greater than that achieved by Medit i700 in both small and large diameters groups. According to our results, Primescan IOS was able to reach the full scanning depth in the large diameter group with the highest level of trueness and precision. Consequently, the manufacturer’s claims were verified. On the contrary, Medit i700 though failed to reach the full scanning depth as avowed by the manufacturer yet, it displayed optimum values of trueness and precision at the depth reached as per the color map.

Accordingly, it’s through the results of that study that we can assure the possibility of chair-side fabrication of a custom-made post with maximum accuracy, even in long post spaces, through the use of the now-available Primescan IOS. A possibility that additionally allows the prosthodontist to avoid the use of physical impressions with all their drawbacks and shortcomings.

However, acknowledging the effect of the oral cavity conditions such as the saliva, as well as the limited accessibility and angle of imaging, the authors would encourage future research to overcome the limitations of the present study and allow in-vivo verification of our results.

## Conclusion

Both the type of IOS as well as the post-space diameter influenced the scan accuracy as well as the depth of the scan. Accuracy results were clearly superior when the Primescan was implemented for scanning the large diameter post space group. Additionally, in the same group, the Primescan was able to successfully record the full depth with similar accuracy to the desktop scanner.

## Electronic supplementary material

Below is the link to the electronic supplementary material.

Supplementary Material 1

## Data Availability

No datasets were generated or analysed during the current study.
