# Supplementary figures and images for: In vitro assessment of the accuracy of two intra-oral scanners for post space scanning in a fully digital workflow
Source: BMC Oral Health. 2025 Mar 19;25:407. doi: 10.1186/s12903-025-05723-x (PMC11924774; doi:10.1186/s12903-025-05723-x)

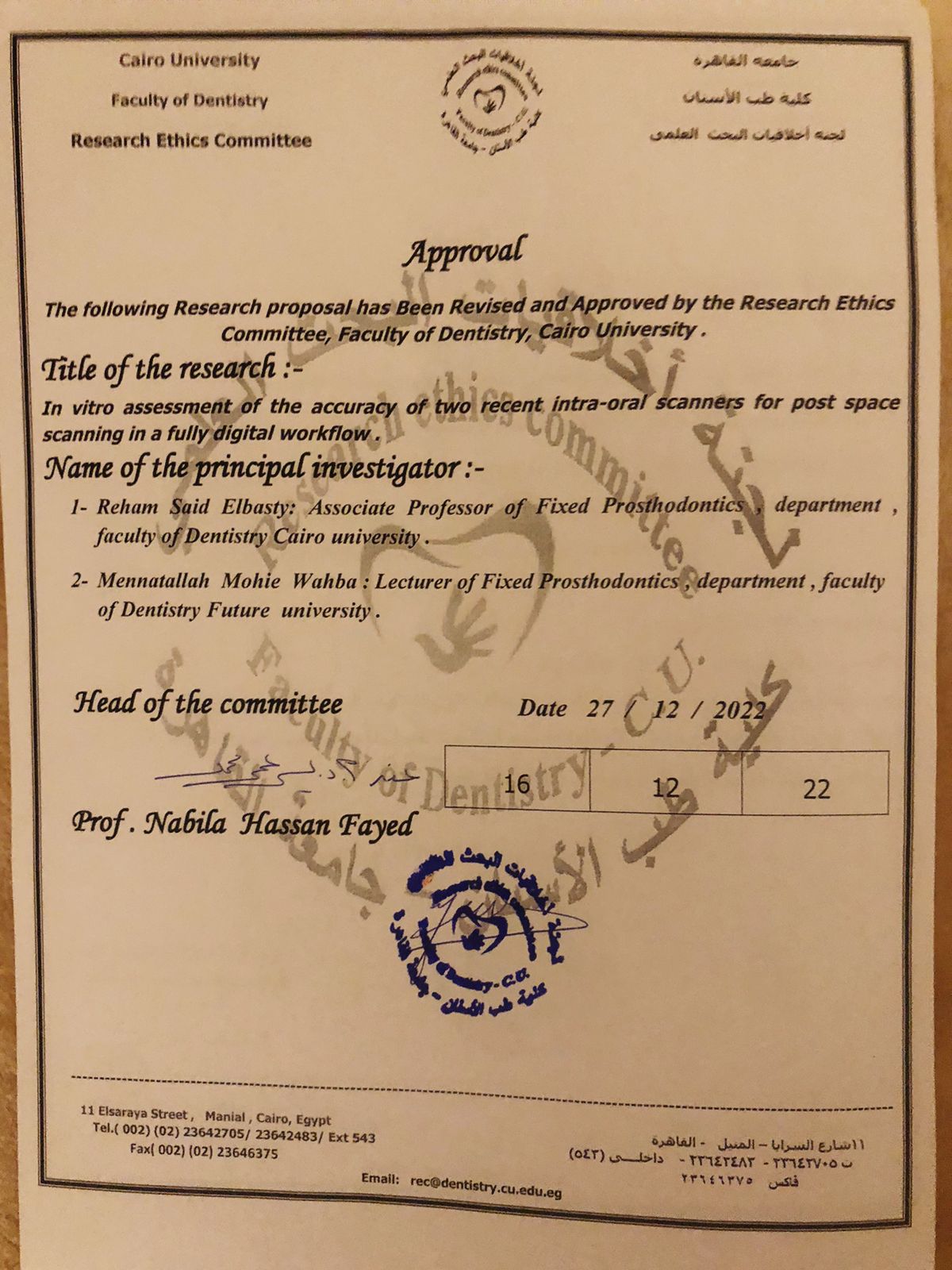

Supplement: Supplementary file 1 — Supplementary Material 1 [file 12903_2025_5723_MOESM1_ESM.jpg]
